# High Brightness Organic Light-Emitting Diodes with Capillary-Welded Hybrid Diameter Silver Nanowire/Graphene Layers as Electrodes

**DOI:** 10.3390/mi10080517

**Published:** 2019-08-03

**Authors:** Jianhua Zhang, Yiru Li, Bo Wang, Huaying Hu, Bin Wei, Lianqiao Yang

**Affiliations:** Key Laboratory of Advanced Display and System Applications, Ministry of Education, Shanghai University, Yanchang Road 149, Shanghai 200072, China

**Keywords:** transparent electrode, silver nanowire, capillary-welded, graphene, organic light-emitting diode

## Abstract

The development of silver nanowire electrodes is always limited due to some disadvantages, such as roughness, oxidative properties, and other disadvantages. In this research, a capillary-welded silver nanowire/graphene composite film was used as an electrode for organic light-emitting diode (OLED) devices. As an encapsulation layer, graphene reduced the surface roughness and the oxidation probability of silver nanowires. The composite electrode showed an excellent transmittance of 91.5% with low sheet resistant of 26.4 ohm/sq. The devices with the silver nanowire/graphene composite electrode emitted green electroluminescence at 516 nm, and the turn-on voltage was about 3.8 V. The maximum brightness was 50810 cd/cm^2^, which is higher than the indium tin oxide-based (ITO-based) devices with the same configuration. Finally, it was proved that the silver nanowire/graphene composite electrodes possessed better heat dissipation than the ITO-based ones under energization. In summary, it means that this novel silver nanowires/graphene electrode has great potential in OLED device applications.

## 1. Introduction

Indium tin oxide (ITO) has a wide range of applications in organic light emitting diode (OLED) devices as transparent electrodes [1]. However, the scarcity of indium reserves has resulted in a high price of ITO electrodes in the market. At the same time, its poor transmission in the near-infrared region cannot meet the needs of transparent devices [2,3]. Therefore, searching for alternative materials of ITO, such as conductive polymers [4,5], carbon nanotubes [6,7], metal arrays [8,9,10], graphene (G) [11,12], and metal nanowires [13], has been one of the important research topics. Among these materials, graphene and metal nanowires stand out and are research hotspots.

Silver nanowire films have been widely studied as electrodes because their transmittance and sheet resistance are comparable to ITO [14,15]. However, the trade balance between optical and electrical properties, the roughness, and oxidation of the silver nanowires limit the development. Welding the silver nanowires is one of the main methods to solve the surface roughness problem [16]. The welding process creates effective connections between silver nanowires and reduces the relative height of the junctions as well [17]. A series of articles about welding methods for silver nanowires have been written [18,19,20], while capillary welding is a green and self-limiting welding method among them [21]. Furthermore, few research articles are about the direction in which welded silver nanowires combine with graphene. Among numerous modified materials, graphene stands out because of its excellent electrical conductivity and the properties to resist water and oxygen [22]. Graphene not only effectively protects silver nanowires from oxidation, but also acts as a conductive layer to increase electrical conductivity. Therefore, a two-dimensional structure electrode composed of silver nanowires with different diameters and graphene was adopted in this research, which are expected to effectively balance the electrical and optical properties of the electrodes, and finally obtain enhanced electrodes. In addition, the research on the optoelectronic device application of these composite electrodes has practical significance [23,24].

In this paper, capillary-welded silver nanowires/graphene electrodes were fabricated for OLED and compared with ITO based devices. A series of characterizations, including transparency, electricity, flatness, stability, and heat dissipation, was carried out to analyze the reason for the performance enhancement of the proposed hybrid electrodes. 

## 2. Materials and Methods

### 2.1. Materials

All chemicals and regents in this work were acquired from commercial sources unless otherwise noted. Graphene was synthesized by chemical vapor deposition (CVD), as described in our previous work [25]. Other organic materials for hybrid device fabrication were obtained from e-Ray Optoelectronics Corp. (Taipei, Taiwan). Silver nanowires with average diameters of 100 nm and 20 nm were purchased from Nanjing XFNANO Materials Tech (Nanjing, China) and Sigma-Aldrich (St. Louis, MO, USA), respectively. 

### 2.2. Preparation of the Silver Nanowire/Graphene Electrode

The glass substrate was washed in acetone, ethanol, and deionized water for 10 min to ensure that the surface was clean enough. Firstly, silver nanowires were spin coated onto the substrate. Silver nanowires were welded using deionized water as a solder to solve the irregularity at line junctions. Thereafter, the CVD-grown graphene was transferred onto the silver nanowire film by the wet transfer method. Finally, the silver nanowire/graphene composite electrode was fabricated on the glass substrate.

### 2.3. Organic Light-Emitting Diode (OLED) Fabrication

The silver nanowire/graphene (AgNW/G) composite electrodes were placed in a high vacuum thermal evaporation chamber, and the OLED configuration was as follows: silver nanowires/graphene/poly (3,4-ethylenedioxythiophene): poly (styrenesulfonate) (PEDOT: PSS)/1,4-bis(N-(1-naphthyl)-N′-phenylamino)-4,4′diamine (NPB): 15 wt% MoO_3_/NPB/4,4′,4″-tris(N-carbazolyl)-triphenylamine (TCTA)/1,3-bis(carbazol-9-yl)benzene (MCP): 10 wt% Ir(ppy)_3_/1,3,5-tris(2-N-phenylbenzimidazolyl)benzene (TPBi)/Liq/Al. Organic layers and the aluminum cathode were deposited on the composite electrode under a high vacuum of 1 × 10^−6^ Pa, whose typical deposition rates were 1.0 nm/s and 5.0 nm/s, respectively. As a comparative experiment, the ITO-based device with the same configuration was also fabricated. 

### 2.4. Film and Device Characterization

The sheet resistance was measured by the Hall Measurement System (ACCENT HL5550LN2, Columbus, OH, United States). The atomic force microscope (AFM, BRUKER, Nasdaq, United States) was applied to characterize the nanostructures of the composite electrodes. Nanostructures were also characterized by a field-emission scanning electron microscope (FE-SEM, Zeiss Sigma 500, Jena, Germany) and the elements were analyzed by energy dispersive spectrometer (EDS, Oxford Ultim max, Oxford, United Kingdom). X-ray photoelectron spectroscopy (XPS, Thermo Fisher Scientific K-Alpha, Massachusetts, United States) was used to test elemental chemical bonds in samples. The current density and luminance versus voltage (J–V–L) characteristics and the electroluminescence (EL) spectra of the OLED devices were measured by a computer-controlled programmable Keithley 2400 source meter (Keithley, Cleveland, OH, United States) and a Photo Research PR 650 spectrometer, respectively. The luminance and spectra of each device were measured in the direction perpendicular to the substrate. The infrared imaging thermometer (FLIR T630sc, Portland, OR, United States) was used to analyze the heat dissipation of these two kinds of electrodes. All measurements were carried out at room temperature under ambient conditions.

## 3. Results and Discussion

**Figure 1:**
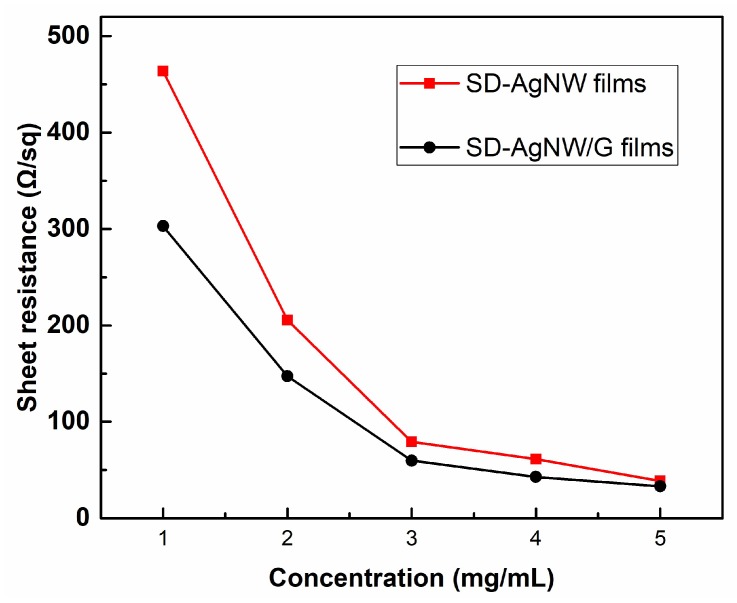
The sheet resistance of the SD-silver nanowire (AgNW) films with different concentrations.

To investigate the electrical properties affected by silver nanowire concentration, the sheet resistance of electrodes was measured, as shown in Figure 1. The figure characterizes the conductive properties of silver nanowires with a diameter of 20 nm (silver nanowires with small diameter, SD-AgNW). For comparison, the sheet resistance of the SD-AgNW/G composite film with a corresponding concentration is also shown in Figure 1. It can be seen that the sheet resistance decreased gradually with the concentration. It proved that the high concentration was more likely to form a denser and more uniform network structure, thereby improving the electrical properties. For the same concentration, the SD-AgNW/G composite electrode exhibited lower sheet resistance compared to the simple silver nanowire film. 

To further optimize properties of the silver nanowire electrodes, another kind of silver nanowire with a diameter of 100 nm (silver nanowires with large diameter, LD-AgNW), which possessed better electrical properties, was introduced to optimize the electrodes. We proposed a novel structure of the electrode, which was made by the spin-coating of silver nanowire solutions of different diameters (silver nanowires with different diameters, SLD-AgNW). LD-AgNW was spin-coated below SD-AgNW, and its larger cross-sectional area made it easier to pass charge carriers. Moreover, the LD-AgNW had a longer length, which effectively reduced the number of wire junctions in the process from one side to the other side of the network. In comparison, the upper SD-AgNW formed denser networks that filled the voids among LD-AgNW. More importantly, electrodes composed of SD-AgNW could collect the charge carriers more effectively because they had a higher effective field. Usually, the figure of merit (FoM = $\sigma_{DC}/\sigma_{OP}$) was used to evaluate the overall performance of the electrode and the corresponding equation was as follows [26]:

(1) $$\frac{\sigma_{DC}}{\sigma_{OP}}=\frac{Z_{0}}{2R_{S}\left(T^{-\frac{1}{2}}-1\right)}$$

where $\sigma_{DC}$ is direct current; $\sigma_{OP}$ is optical conductivity; and $R_{S}$ and T are sheet resistance and transmittance, respectively. By comparing the data in Table 1, the FoM of LD-AgNW electrodes (42.9) was slightly higher than SD-AgNW electrodes (59.4), which were both lower than SLD-AgNW electrodes (91.9). 

**Figure 2:**
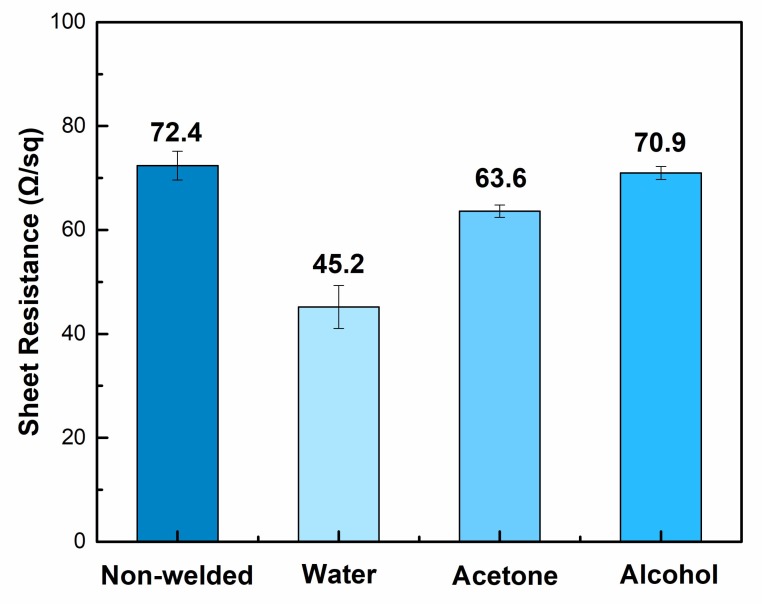
The sheet resistance of SLD-AgNW films welded with different liquids.

The wire junction problem is one of the most significant factors affecting the quality of silver nanowire electrodes. Poor contact at the wire junctions not only caused non-conduction among wires, but also increased the surface roughness, which ultimately led to high current leakage of the devices [27]. Capillary welding is considered to be an environmentally effective welding method [21]. Its self-limitation determines that welding will not damage the network themselves. In this paper, different liquids, including deionized water (DI water), acetone, and alcohol, were tried as soldering agents to weld the SLD-AgNW networks. According to the calculation formula [20], the capillary force is proportional to the surface tension (γ) of the soldering agents. At room temperature, the surface tension of water is 72.8 mN/m, which is much higher than acetone (24.0 mN/m) and alcohol (21.8 mN/m). In theory, when DI water was used as a soldering agent, the capillary force generated at the liquid bridge was the largest, so the welding effect was the most significant. Figure 2 shows the sheet resistances of SLD-AgNW films welded with different liquids, and the results of each group displayed the data of five samples. The welding effect of alcohol soldering agents was not obvious, and the sheet resistance indeed decreased after welding by acetone and DI water. In particular, the sheet resistance of SLD-AgNW networks welded by DI water was reduced from 72.38 Ω/sq to 45.18 Ω/sq, improving the electrical properties of transparent electrodes remarkably. At the same time, DI water is an environmentally friendly soldering agent that will not introduce any impurities into the sample. Therefore, DI water was used to weld the SLD-AgNW networks as a welding agent.

After welding with DI water, SLD-AgNW were tightly crimped together, and the effective connection was established at the junctions to further improve the electrode performance. At the same time, the surface roughness was reduced to 7.4 nm, which is crucial for the subsequent process of fabricating the device. Finally, the FoM of the welded SLD-AgNW electrodes was increased to 145, and it reached 157 for the corresponding silver nanowire/graphene composite electrodes (Table 1).

**Figure 3:**
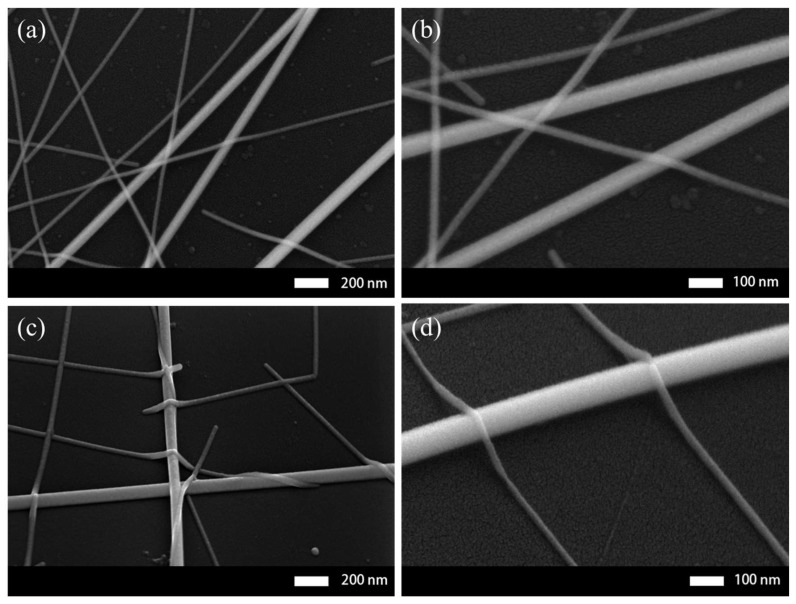
(**a**) Low-and (**b**) high-magnification scanning electron microscope (SEM) images of the SLD-AgNW network without welding. (**c**) Low- and (**d**) high-magnification SEM images of the welded SLD-AgNW network.

SEM images were used to verify the effect of capillary force welding by characterizing the surface morphology of the composite film. Figure 3a and b show SEM images of the SLD-AgNW film without welding. As can be seen from Figure 3a, the SD-AgNW were stacked over the LD-AgNW with only slight deformation. Figure 3b is an enlarged view of Figure 3a, where significant voids between nanowires were seen. These voids indicated that few effective connections were made between silver nanowires, which affected the transfer efficiency of charge between the nanowires. In contrast, Figure 3c,d show welded films with the SLD-AgNW in which the above SD-AgNW were severely deformed. Furthermore, within a large area, almost all of the nanowires were tightly connected together at the wire junction locations. Figure 3d shows more clearly the effective connection between SD-AgNW and LD-AgNW. The upper SD-AgNW were embedded in the below LD-AgNW, which not only provided more channels for charge transfer, but also effectively reduced the height of the junctions to reduce the surface roughness.

**Figure 4:**
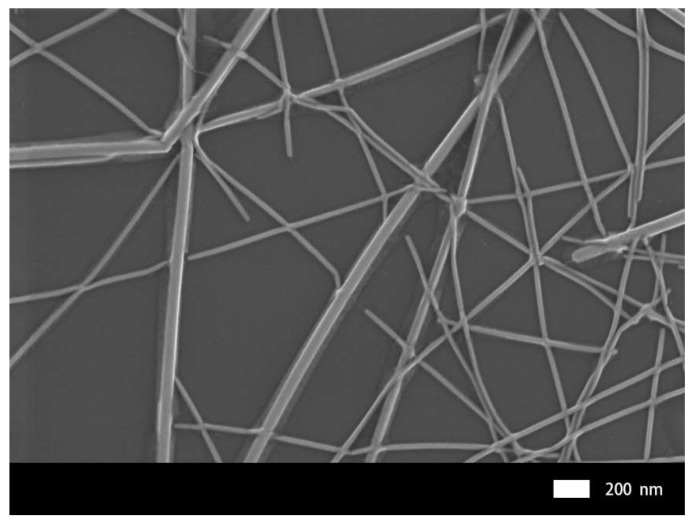
SEM image of SLD-AgNW/graphene films.

**Figure 5:**
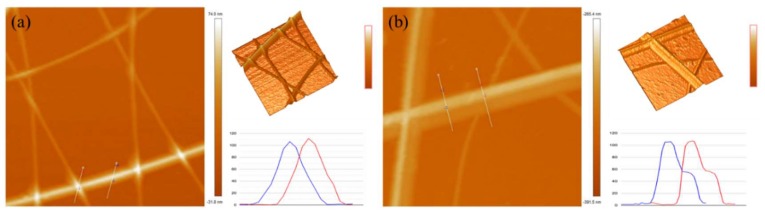
The atomic force microscope (AFM) images of (**a**) SLD-AgNW films and (**b**) SLD-AgNW/G films. The images at the upper right show the 3D AFM images, and the images at the bottom right show the height contrast between the line junctions and the line level.

The surface roughness of electrodes plays a dominant role in the fabrication of optoelectronic devices. Irregularities and protrusions on the surface will eventually create high leakage currents due to short circuits in the devices. The SEM image of SLD-AgNW/G film is shown in Figure 4. It can be seen that graphene was uniformly tiled on the SLD-AgNW network, and there were no obvious breakages or wrinkles of graphene. The uniform coverage of graphene may lead to the reduction of surface roughness [19]. In order to further quantify the variation of surface roughness, AFM images were captured for the SLD-AgNW films and the SLD-AgNW/G composite films, and the results are shown in Figure 5. The RMS of SLD-AgNW networks was 7.4 nm, and it was further reduced to 6.4 nm for the composite films. The images at the upper right show three-dimensional AFM images of the electrodes. By comparison, it was obvious that the composite film was smoother with lower surface protrusion height. The image at the bottom right shows the height contrast between the line junctions and the line level. After being compounded with graphene, the height was negligible, which greatly improved the roughness problem of the silver nanowire electrodes.

**Figure 6:**
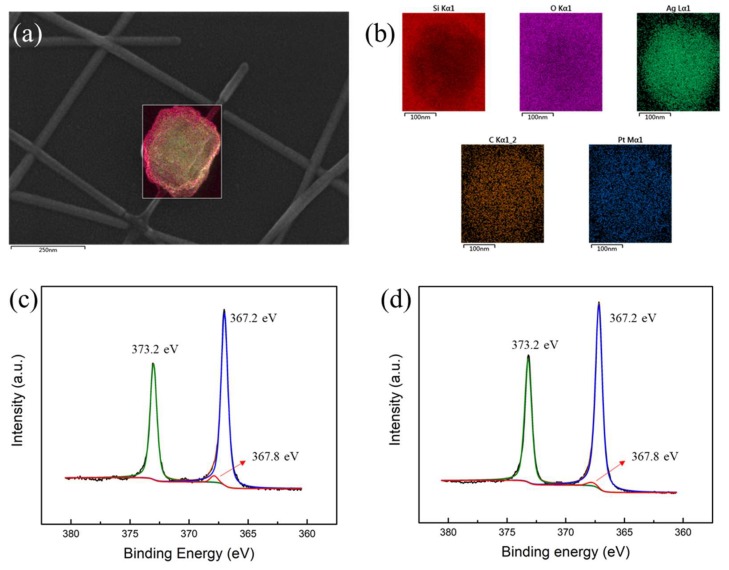
(**a**) The SEM image and (**b**) the element analysis of unknown particles in silver nanowire films. XPS spectra of Ag 3d in (**c**) SLD-AgNW films and (**d**) SLD-AgNW/G films after being annealed in the atmosphere at 120 °C for 12 h.

Graphene has the function of isolating water vapor, which can effectively protect the silver nanowire from being oxidized. There were some particles on the silver nanowire films, whose morphology is shown in Figure 6a. In order to determine the composition of the particles, we performed the EDS test. Figure 6b shows the elemental distribution of the particles, and the detected elements included Si, O, Ag, C, and Pt. XPS was chosen to further detect the valence state of the elements (Figure 6c,d). There were three main peaks in the XPS, with the position of 373.4 eV, 367.4 eV, and 367.8 eV. This meant that Ag existed in the form of Ag 3d_3/2_ (373.4 eV) and Ag 3d_5/2_ (367.4 eV). At the same time, the peak of 367.8 eV represented Ag_2_O, which indicated that part of the silver nanowires was oxidized in the SLD-AgNW films [28]. Obviously, compared with the SLD-AgNW films, the peak intensity at 367.8 eV was significantly reduced in the SLD-AgNW/G films (Figure 6d). This strongly demonstrates that the encapsulation of graphene effectively prevents oxidation of the silver nanowires.

**Figure 7:**
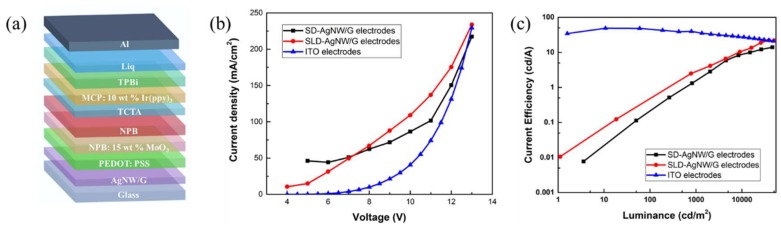
(**a**) The configuration of the organic light-emitting diode (OLED) devices with silver nanowire/graphene electrodes. (**b**) Current density–voltage characteristics and (**c**) current efficiency-luminance of OLED devices with indium tin oxide (ITO), SD-AgNW/G, and SLD-AgNW/G film as anodes.

To further evaluate the practical application potential of the electrodes, a series of OLED devices was fabricated, the configuration of which was AgNW/G/PEDOT:PSS/NPB:15 wt% MoO_3_ (100 nm)/NPB(30 nm)/TCTA (10 nm)/ MCP:10 wt% Ir(ppy)_3_ (20 nm)/TPBi (40 nm)/Liq (1 nm)/Al (150 nm) (Figure 7a). The OLED device was composed of PEDOT: PSS as the hole-injection buffer layer, both NPB (including the NPB: 15 wt% MoO_3_) and TCTA as the hole-transporting layer, MCP: 10 wt% Ir(ppy)_3_ as the emitting layer, TPBi as the electron-transporting layer, Liq as the electron-injecting layer and Al as the cathode. After energization, electrons and holes are injected from the cathode and the anode to the light-emitting layer, respectively. The injected electrons and holes migrate from the electron transport layer and the hole transport layer. After electrons and holes are injected into the light-emitting layer, they are bound together by the action of Coulomb force to form excitons. The exciton radiation transition emits photons and releases energy. Homomorphic devices with ITO as the anode were fabricated as a comparative experiment. The device, using only the silver nanowires as the electrode, quenched quickly after being energized, which may be due to the high leakage current caused by the unevenness of the silver nanowires. The same situation also occurred in the LD-AgNW/G based devices, which further illustrated the optimization necessity of electrode structures. As shown in Figure 7b, the SLD-AgNW/G devices and ITO devices were turned on at about 3.8 V, while the turn-on voltage of SD-AgNW/G devices was slightly higher (about 5 V). The main reason was that the lower sheet resistance of SLD-AgNW/G electrodes enables efficient injection of carriers from the electrode to the organic functional layer. The CE_max_, PE_max_, and EQE_max_ of the OLED devices were 22.20 cd/A, 5.81 lm/W, 6.58%, respectively. The current efficiency-luminance (CE-L) characteristics for OLEDs are illustrated in Figure 7c. There was a slight decrease in the current efficiency of the ITO based devices, while for both SD-AgNW/G and SLD-AgNW/G based devices, the current density increased progressively with brightness. Besides, at the same brightness, the current density of the SLD-AgNW/G devices were always higher than that of the SD-AgNW/G devices. Moreover, in the case of high brightness, the current efficiency of the SLD-AgNW devices was briefly higher than that of the ITO devices.

**Figure 8:**
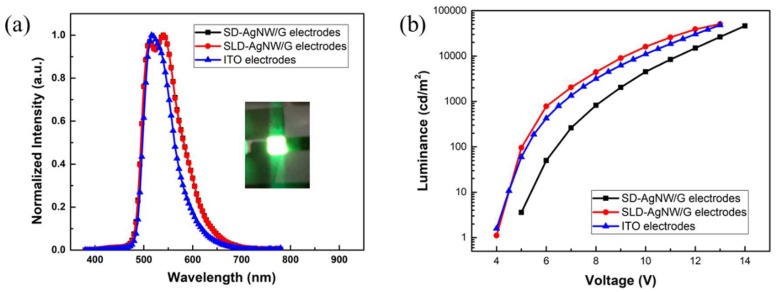
(**a**) The spectrum and (**b**) luminance–voltage characteristics of OLED devices with ITO, SD-AgNW/G and SLD-AgNW/G film as anodes.

The EL properties of OLED devices are shown in Figure 8a. The actual illumination image of the SLD-AgNW/G devices, as shown in the inset image, clearly showed that the devices emitted green light, which has a peak wavelength of 516 nm with a minor shoulder at 540 nm. The reason for the difference of the spectrum may be due to the higher refractive index of the silver nanowire resulting in a stronger microcavity effect. As shown in Figure 8b, another advantage of the SLD-AgNW/G-based devices was that they achieved higher brightness with a maximum brightness of 50810 cd/cm^2^ compared to ITO devices (48050 cd/cm^2^). The brightness of SD-AgNW/G devices was relatively low, which illustrated the potential and advantages of the SLD-AgNW/G electrodes in device applications.

**Figure 9:**
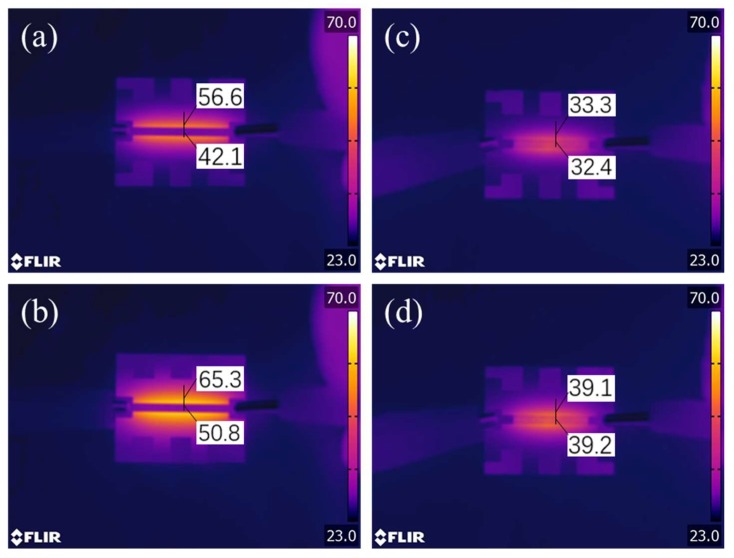
Temperature distribution profile of the electrodes operated at 150 mA/cm^2^ for 30 s (upper) and 60 s (below) for ITO (**a**,**b**) and SLD-AgNW/G (**c**,**d**), respectively.

The temperature and heat dissipation capabilities of OLED devices have a huge impact on their performance. OLED devices generate a large amount of heat during operation due to the relatively low optical efficiency, and excellent thermal conductivity can reduce device failure under long-term operation [29]. Since the silver nanowire/graphene electrodes possess better heat dissipation ability, they effectively and quickly dissipate the generated heat so that the devices can work normally at high electric power to obtain higher luminance. The accumulation of heat at the electrodes also accelerates the aging of the organic functional layer, severely reducing device life. Here, a near-infrared camera was used to observe the heat dissipation performance of the electrode under energization, and the current was set to 150 mA/cm^2^. As seen in Figure 9a,b, after 30 s of energization, the temperature rise of the ITO electrode was obvious, and the center temperature and the side temperature were 42.1 °C and 56.6 °C, respectively. Furthermore, during the process of increasing the energization time to 60 s, the temperature of the two points continued to rise and finally reached 50.8 °C and 65.3 °C, respectively. In contrast, silver nanowire/graphene electrodes exhibited superior thermal performance. After energization for 60 s, its center temperature and side temperature were still much lower than those of ITO. As shown in Figure 9c,d, the center temperature was 39.1 °C, and the side temperature was 39.2 °C. The above results indicated that the SLD-AgNW/G electrodes have great potential as alternative electrodes for optoelectronic devices.

## 4. Conclusions

In conclusion, we fabricated OLED devices using capillary-welded silver nanowire/graphene composite films as electrodes. The composite electrode showed an excellent transmittance of 91.5% with low sheet resistant of 26.4 ohm/sq. The OLEDs with welded hybrid diameters silver nanowire/graphene films as electrodes exhibited the highest brightness. Furthermore, the maximum brightness was 50810 cd/cm^2^, which is higher than the indium tin oxide-based (ITO-based) devices with the same configuration. The devices emitted green electroluminescence at 516 nm, and the turn-on voltage was about 3.8 V. Furthermore, it proved that the silver nanowire/graphene composite electrodes possessed better heat dissipation than the ITO-based ones under energization. 

## Figures and Tables

**Table 1:** The sheet resistance, transmission, and figure of merit (FoM) of different electrodes.

Electrode Composition	Sheet Resistance (ohm/sq)	Transmission (%)	FoM
**SD-AgNW**	302.9	98.4	59.4
**LD-AgNW**	38.6	80.6	42.9
SLD-AgNW	71.5	94.5	91.9
Welded SLD-AgNW	45.2	94.5	145.2
Welded SLD-AgNW/graphene (SLD-AgNW/G)	26.4	91.5	157.0

## Data Availability

The date generated during and/or analyzed during the current study are available from the corresponding authors on reasonable request.
